# Identification of a metabolic brain network characterizing essential tremor

**DOI:** 10.1038/s41598-024-82069-4

**Published:** 2025-01-16

**Authors:** Solange Volnov, Hamzah Baagil, Oliver Winz, Hans-Juergen Kaiser, Sanne Katherina Meles, Joerg Bernhard Schulz, Kathrin Reetz, Felix Manuel Mottaghy, Florian Holtbernd

**Affiliations:** 1 Department of Neurology, University Hospital RWTH Aachen, Pauwelsstr. 30, 52074 Aachen, Germany; 2 Department of Nuclear Medicine, University Hospital RWTH Aachen, Aachen, Germany; 3 Department of Neurology, University Medical Centre Groningen, University of Groningen, Groningen, The Netherlands; 4 JARA-BRAIN Institute Molecular Neuroscience and Neuroimaging, Forschungszentrum Juelich GmbH, Rheinisch-Westfaelische Technische Hochschule Aachen University, Aachen, Germany; 5 Department of Radiology and Nuclear Medicine, Maastricht University Medical Center (MUMC+), Maastricht, The Netherlands; 6 Institute of Neuroscience and Medicine (INM-4/INM-11), Forschungszentrum Juelich GmbH, Juelich, Germany

**Keywords:** Deep brain stimulation, Essential tremor, FDG PET, Brain metabolism, Spatial covariance analysis, Motor control, Neural circuits, Network models, Neuroscience, Neurodegeneration

## Abstract

The neuronal correlate of tremor genesis and cognitive function in essential tremor (ET) and its modulation by deep brain stimulation (DBS) are poorly understood. To explore the underlying metabolic topography of motor and cognitive symptoms, sixteen ET patients (age 63.6 ± 49.1 years) and 18 healthy controls (HC) (61.1 ± 6.3 years) underwent tremor and cognitive assessments and^18^F-fluorodeoxyglucose PET of the brain. Multivariate spatial covariance analysis was applied for identifying ET related metabolic brain networks. For network validation and to explore DBS effects, 8 additional ET patients (68.1 ± 8.2 years) treated with DBS were assessed in both the ON and OFF state, respectively. The ET related metabolic spatial covariance pattern (ETRP) was characterized by relatively increased metabolism in the cerebellum, brainstem, and temporo-occipital cortices, accompanied by relative metabolic decreases mainly in fronto-temporal and motor cortices. Network expression showed inverse correlations with tremor severity and disease duration and positive correlations with cognitive dysfunction. DBS substantially alleviated tremor, but had only marginal effects on cognitive performance. There were no significant DBS effects on ETRP expression at the group level, but all but one subject showed higher scores in the ON state. Our findings suggest ET is characterized by an abnormal brain network associated with disease phenotype.

**Supplementary Information:**

The online version contains supplementary material available at 10.1038/s41598-024-82069-4.

## Introduction

Essential tremor (ET) is among the most common movement disorders in adulthood^1^. The defining clinical hallmark is an action tremor of the hands^2^. The pathophysiology underlying essential tremor still is poorly understood. The early concept of ET postulated a primary role of the inferior olive in tremor genesis^3^. However, there is mounting evidence from structural and functional imaging studies suggesting that tremor originates from an oscillatory network in which the cerebellum occupies a key role^4^.

It is increasingly recognized that cognitive impairment is common in ET patients^5^. People with ET are at increased risk to develop mild cognitive impairment or even overt dementia^5^. There is no established neuropsychological profile characterizing cognitive impairment in ET, but several studies have reported deficits mainly in executive, attention, and verbal memory domains that partially overlap with those observed in Parkinson’s disease (PD)^6–8^. In line with studies on tremor genesis, recent work indicates that cognitive impairment in ET is caused by network level brain changes that include disruptions of cerebello-cortical connectivity, but also involve alterations outside the classic tremor network^7,9,10^.

Whereas a large body of structural and functional MRI studies explored ET related brain changes, few^18^F-fluorodeoxyglucose (FDG) positron emission computed tomography (PET) studies have explored quantitative metabolic brain activity in ET^11^. These studies applied region of interest or univariate voxel based approaches to explore regional metabolic ET related brain changes^11^. Metabolic imaging studies using a network level approach to characterize the complex brain changes associated with ET are lacking. To this end, the scaled subprofile model principal component analysis (SSM/PCA) has been tailored to identify disease specific spatial covariance patterns in metabolic imaging data of combined samples of patient and control data^12^. SSM/PCA based analyses do not require a priori assumptions regarding the contribution of specific brain regions to a given pattern. Furthermore, analyses are blind to subjects’ group assignments and largely data driven. SSM/PCA has successfully been utilized to identify highly specific covariance patterns in PD and other neurodegenerative diseases^12^.

Here, we sought to (1) identify a whole brain ET related metabolic spatial covariance pattern (ETRP) that reliably distinguished ET patients from healthy controls (HC); to (2) investigate the relationship of network expression with tremor severity and cognitive function; to (3) explore modulatory effects of deep brain stimulation (DBS) of the ventral intermediate nucleus of thalamus, a powerful therapy to alleviate tremor in severely affected subjects^13^, on ETRP expression, and finally, (4) to investigate the relationship of network level metabolic brain changes in ET with those observed in PD (PD related pattern (PDRP))^14^.

## Methods

### Subjects

Sixteen patients with essential tremor (ET1, age 63.6 ± 9.1 years, 8 females) were recruited through the out-patient clinic of the department of Neurology at RWTH Aachen University, Germany. The diagnosis was made by an experienced movement disorders specialist (FH) according to established criteria^2^. None of the patients included had a prior history of drug abuse, concomitant neurological-psychiatric disease, or severe metabolic or cardiac disorder. Seven patients of the ET1 cohort received ET treatment with propranolol (*n* = 3, daily dose: 120 ± 69.3 mg) or primidone (*n* = 6, daily dose: 285 ± 284.1 mg). One of these subjects received both propranolol and primidone, another subject on primidone was additionally treated with 200 mg q.d. of gabapentin. For comparison, eighteen age and gender matched healthy controls (HC, age 61.1 ± 6.3, 9 females) were recruited. For validation of network analysis and to explore the effect of DBS on behavioral and imaging measures, we recruited a second, independent ET cohort comprised of eight subjects (ET2 age 68.1 ± 8.2 years, 4 females). These patients underwent DBS of the ventral intermediate nucleus of thalamus at least 3 months (65.1 ± 72.6 months) prior to study inclusion. One of these subjects underwent unilateral (left-sided) DBS, all other subjects received bilateral electrodes. The inclusion criteria for ET2 subjects were the same as for ET1 individuals. ET2 subjects underwent clinical assessments and FDG-PET imaging twice, with the DBS system turned ON and OFF, respectively. For testing in the OFF state, the stimulator was turned off 12 h prior to testing. The time span between the scans with the DBS turned ON and OFF averaged 4.75 ± 1.58 days. Patients receiving ET specific medication were scanned on their regular dose.

All participants provided written informed consent prior to study inclusion. The study was conducted according to the declaration of Helsinki and was approved by the local ethics committee at the medical faculty of RWTH Aachen University (EK 236 − 15) and The German Federal Office of Radiation Protection (Z5- 22464/2016-002-S/G).

### Clinical and cognitive assessments

All subjects underwent routine clinical examination to exclude the presence of neurological symptoms but those attributable to ET. Particular care was taken to make sure no subjects revealing subtle signs of parkinsonism were included. Clinical examination was unrevealing in all HC included. Tremor severity was assessed using the Fahn-Tolosa Marin-Scale (FTM)^15^. All participants were subjected to detailed neuropsychological testing, including stroop color and word test (STROOP^16^), California verbal learning test (CVLT^17^), trail making test (TMT^18^), brief test of attention (BTA^19^), digit span test (DGS^20^), Regensburger Wortfluessigkeits-Test (RWT^21^) (test of verbal fluency), Leistungspruefsystem (LPS^22^) (test of global cognitive capability), Mini-Mental State Examination (MMSE^23^), Montreal Cognitive Assessment (MOCA^24^), Block Design Test^25^, and Complex Figure Test^26^. The presence of depression was assessed using the Beck depression inventory (BDI^27^). Due to severe tremor, some ET subjects were not able to perform all neuropsychological tasks.

### FDG-PET imaging

All participants underwent FDG-PET imaging on a Siemens ECAT ECAT HR + whole body scanner in three-dimensional mode (Siemens Healthcare, Erlangen, Germany). All scans were performed under resting conditions with the eyes open and ambient noise. The patient’s head was positioned in a head shell and secured with an elastic band. FDG-PET scans were obtained 40 min after an intravenous injection of 207 ± 23 MBq of FDG. All patients fasted for at least 6 h before tracer injection and plasma glucose levels were within the reference range. Four emission images of 5 min each were recorded with previously measured attenuation correction (10-min 68Ge/68Ga transmission scan). Slices (*n* = 63; thickness, 2.425 mm; pixel size, 2 × 2 mm) were reconstructed per time frame by filtered backprojection (Hanning filter, FWHM 4.0 mm; zoom, 3). All datasets were fully corrected for scatter radiation and attenuation and only the summed PET datasets (20-min total scan time) were used for further analyses.

### Data processing

FDG-PET images were preprocessed in SPM 12 (Wellcome Department of Imaging Neuroscience, Institute of Neurology, London, UK) implemented in MATLAB (version R2020b; MathWorks, Natick, Massachusetts). Images were normalized to Montreal Neurologic Institute (MNI) standard space using a previously published reference template^28^, and smoothed using an 8 mm FWHM kernel.

### Network identification

We applied SSM/PCA to identify an ET related metabolic pattern (ETRP)^29^. Code from the University Medical Center Groningen^29^ based on the method by Spetsieris and Eidelberg^30^ implemented in MATLAB was used to perform SSM-PCA analyses. In brief, images were masked based on an intensity threshold of 35% of the whole-brain maximum, which corresponds to mainly gray matter. After log transformation of masked images, subject means were removed. Next, the mean value across HC was removed from each subject’s scan for off-setting. A PCA was performed on these residual images in voxel space. Only those PCs explaining the top 50% of variance were considered for further analyses. Scores for selected PCs were computed for each scan and entered into a logistic regression model. One or more PCs best discriminating HC from ET patients were linearly combined to create the ETRP. Each PC contributing to the ETRP was weighted using the corresponding coefficient from the logistic regression model. Bootstrap validation using 1000 iterations at a 95% confidence interval was applied to validate pattern topography^29,31^. Only HC and ET1 subjects were used for pattern derivation. The bootstrapped pattern was superimposed on a standard T1 MRI template for visualization. A previously identified pattern can be used to quantify the FDG-PET scans of new subjects. In this procedure, an individual’s scan is projected onto the pattern, resulting in a single score (individual pattern expression)^29^. Thus, pattern expression was prospectively computed in ET2 individuals to validate disease specificity of the ETRP. For comparison of ET2 subject scores with ET1 and HC groups, network scores of ET1 and ET2 subjects were z-transformed with respect to HC, so that the mean of the HC group equalled 0 with a SD of 1.

### Voxel weight correlations of ETRP and PDRP

To explore the relationship of the ETRP with PDRP topography, we cross-correlated ETRP and PDRP voxel weights by computing the Pearson product-moment correlation coefficient. Non-zero voxels from each pattern image (within a common mask) were formatted into a single vector. The two vectors were then entered into the MATLAB statistical routine ‘corr’ to calculate the correlation coefficient. In addition, we calculated individual PDRP expression in each HC, ET1, and ET2 scan. We utilized a previously published PDRP version from a Dutch PD cohort for all computations^14^.

### Statistical analyses

We used Shapiro-Wilk and Kolmogorov-Smirnov tests to test for normality of the data. ANOVA with post-hoc Bonferroni tests, Kruskall-Wallis tests with Bonferroni corrected post-hoc Dunn’s tests, Student’s t-tests, and Chi-square tests were applied to compare demographical, clinical and neuropsychological measures, and ETRP/PDRP expression among groups as appropriate. For within subject comparisons in the ET2 group (ON vs. OFF), we utilized the paired t-test and Wilcoxon signed rank test, respectively. The Pearson correlation coefficient or Spearman correlation coefficient were computed for correlational analyses of network scores with behavioral measures as appropriate. A p-value < 0.05 was deemed significant. All analyses were performed in IBM SPSS Statistics (Version 27).

## Results

### Demographic and clinical data

Age and gender did not differ among groups (age: F(2,39) = 2.231, *p* = 0.121; gender: χ^2^ (2) = 0.00, *p* = 1.00). The level of education (years of education) did not differ among groups (F (2,38) = 1.983, *p* = 0.152). There was no significant difference of disease duration between ET1 and ET2 subjects (F(1,22) = 3.098, *p* = 0.092; Student’s t-test). FTM scores differed across groups (F(2,33) = 39.046, *p* < 0.001). Post-hoc tests revealed higher scores in both ET1 (*p* < 0.001) and ET2 cohorts (*p* < 0.001) compared with HC. ET2 subjects (OFF state) were more severely affected than their ET1 counterparts (*p* < 0.001). Demographic and clinical data are summarized in Table 1.

**Table 1:** Demographics and clinical data. Values are presented as mean ± SD. FTM = Fahn-Tolosa-Marin Scale; n.a.: not applicable; a: ambidextrous; r: right-handed. ^**§**^One-way ANOVA/Student’s t-test/Chi-square test; significant results are printed in *italics*. *missing in 1 subject; ^**^missing in 6 subjects; ^&^post-hoc Bonferroni tests (HC vs. ET1; HC vs. ET2; ET1 vs. ET2; *p* < 0.001 for all).

	HC	ET1	ET2	*p*-value^§^
**N**	18	16	8	
**Gender (f/m)**	9/9	8/8	4/4	1.00
**Age (y)**	61.1 ± 6.3	63.6 ± 9.1	68.1 ± 8.2	0.121
**Education (y)**	11.7 ± 1.7	10.9 ± 1.9	10.1* ± 2.1	0.152
**Age of onset (y)**	n.a.	41.2 ± 23.1	32.0 ± 19.6	0.348
**Disease duration (y)**	n.a.	22.4 ± 17.9	36.1 ± 17.9	0.092
**FTM score**	0.9 ± 1.8^**^	26.6 ± 13.5	54.9 ± 21.4	*< 0.001* ^&^
**Handedness (a/r)**	(0/18**)	(1/15)	(0/8)	0.526

### Neurocognitive performance

**Fig. 1:**
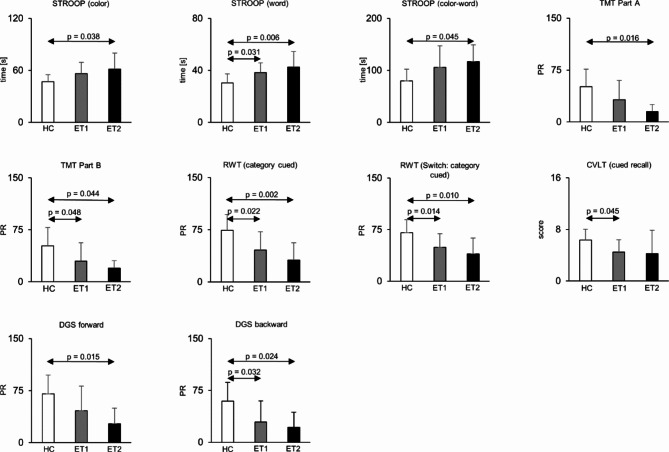
Cognitive performance across groups. STROOP: Stroop test (color: color naming, word: word reading, color-word: color-word interference task); TMT = Trail making test; RWT = Regensburger Wortfluessigkeitstest; CVLT = California verbal learning test; DGS = digit span test; PR = Percentile rank. Bars present group means, error mars indicate the standard deviation.

ET subjects performed worse compared with HC on several tests, mainly relating to executive function, working memory, and verbal fluency. Specifically, post-hoc group-wise comparisons of significant ANOVA and Kruskall-Wallis tests revealed lower performance in ET subjects compared with HC for the STROOP (word: ET1 vs. HC: *p* = 0.031, ET2 vs. HC: *p* = 0.006; color: ET2 vs. HC: *p* = 0.038; color-word: ET2 vs. HC: *p* = 0.045), both the category-cued and category-cued switching tasks of the RWT (ET1 vs. HC: *p* = 0.022, ET2 vs. HC: *p* = 0.002 and ET1 vs. HC: *p* = 0.014, ET2 vs. HC: *p* = 0.010), cued-recall task of the CVLT (ET1 vs. HC: *p* = 0.045), DGS forward (ET2 vs. HC: *p* = 0.015) and backward test (ET1 vs. HC: *p* = 0. 032, ET2 vs. HC: *p* = 0.024), and TMT part A (ET2 vs. HC: *p* = 0.016) and part B (ET1 vs. HC : *p* = 0.048, ET2 vs. HC : *p* = 0.044)(Fig. 1). We did not find any differences of cognitive performance between ET1 and ET2 cohorts (*p* > 0.44). Depressive symptoms as assessed by the BDI were higher in ET1 subjects compared with both HC and ET2 subjects (ET1 vs. HC: *p* = 0.006, ET1 vs. ET2: *p* = 0.025). Importantly, BDI scores were not correlated with cognitive performance in any of the tests in the overall ET cohort (*p* > 0.09). There was an inverse correlation between FTM scores and the category-cued subtest of RWT (*p* = 0.034; *r* = -0.434). No further correlations were observed between tremor severity and cognitive performance (*p* > 0.05). The full descriptive and test statistics are summarized in Tables 2 and 3, respectively.

**Table 2:** Descriptives of neuropsychological tests. MOCA = Montreal cognitive assessment; MMSE = Mini mental state examination; CVLT = California verbal learning test; TMT = Trail making test; word = word reading; color = color naming; color-word = color-word interference; LPS = Leistungspruefsystem; BTA = brief test of attention; RWT = Regensburger Wortfluessigkeitstest; BDI: Beck’s depression index; PR = percentile rank. Values are presented as mean ± SD (number of subjects).

Domains	Test	Mean ± SD (*n*)		
		HC	ET1	ET2
**Global**	**MOCA [score]**	26.0 ± 2.8 (17)	24.1 ± 2.8 (16)	24.0 ± 3.2 (5)
	**MMSE [score]**	27.8 ± 1.7 (18)	27.0 ± 1.6 (16)	28.2 ± 1.1 (5)
**Memory**	**CVLT [score]**			
	**Immediate recall**	26.7 ± 4.3 (18)	22.9 ± 4.9 (15)	22.5 ± 6.5 (8)
	**Short delay recall**	7.1 ± 1.3 (18)	5.8 ± 1.5 (15)	6.4 ± 2.0 (8)
	**Long delay recall**	6.4 ± 1.9 (18)	4.7 ± 2.1 (15)	4.8 ± 3.3 (8)
	**Cued recall**	6.3 ± 1.7 (18)	4.5 ± 2.0 (15)	4.3 ± 3.8 (8)
	**Recognition**	25.9 ± 1.6 (18)	25.5 ± 1.1 (15)	25.3 ± 1.4 (8)
**Executive function**	**TMT [PR]**			
	**Part A**	51.0 ± 26.2 (18)	31.9 ± 28.8 (16)	15.1 ± 10.7 (7)
	**Part B**	51.9 ± 27.3 (18)	29.8 ± 27.4 (16)	19.7 ± 11.6 (7)
	**Stroop [sec]**			
	**Word**	30.3 ± 7.1 (18)	38.3 ± 7.8 (16)	42.5 ± 12.8 (8)
	**Color**	46.9 ± 8.3 (18)	56.3 ± 13.4 (16)	61.4 ± 19.8 (8)
	**Color-word**	79.7 ± 23.3 (18)	105.5 ± 43.0 (16)	116.6 ± 34.6 (8)
**Visuospatial** **function**	**LPS [score]**			
	**Subtest 7**	15.9 ± 7.6 (17)	12.6 ± 6.6 (16)	11.9 ± 4.0 (7)
	**Subtest 9**	22.6 ± 6.6 (18)	18.2 ± 4.6 (16)	18.9 ± 3.9 (7)
	**Block design [T-value]**	54.1 ± 12.9 (18)	45.2 ± 8.1 (16)	44.0 ± 7.2 (5)
	**Complex Figure Test [score]**			
	**Copy**	35.5 ± 0.9 (18)	34.8 ± 1.6 (16)	35.5 ± 1.0 (4)
	**Delayed**	17.6 ± 7.9 (18)	16.8 ± 5.2 (16)	22.6 ± 10.4 (4)
**Working memory** **and attention**	**Digit span [PR]**			
	**Forward**	70.1 ± 27.9 (18)	46.2 ± 36.5 (16)	27.4 ± 23.7 (8)
	**Backward**	59.6 ± 27.9 (18)	29.4 ± 31.4 (16)	21.5 ± 23.6 (8)
	**BTA [T-value]**	50.8 ± 10.7 (18)	46.5 ± 12.1 (16)	44.6 ± 6.0 (8)
**Verbal fluency**	**RWT [PR]**			
	**Letter-cued**	64.6 ± 29.2 (18)	48.2 ± 26.3 (16)	44.1 ± 26.9 (8)
	**Switching**: **letter-cued**	62.0 ± 26.6 (18)	40.9 ± 31.7 (16)	43.8 ± 26.6 (8)
	**Category-cued**	74.3 ± 23.4 (18)	45.9 ± 27.1 (16)	31.4 ± 26.7 (8)
	**Switching: category- cued**	70.6 ± 19.3 (18)	49.3 ± 20.2 (16)	39.8 ± 24.7 (8)
**Mood**	**BDI [score]**	4.7 ± 4.7 (18)	11.8 ± 8.1 (16)	4.3 ± 4.3 (8)

**Table 3:** Results of neuropsychological tests. Significant results are printed in *italics*. HC: healthy controls; ET1: essential tremor cohort 1; ET2: essential tremor cohort 2. ET2 subjects were assessed off stimulation. MOCA = Montreal cognitive assessment; MMSE = Mini mental state examination, CVLT = California verbal learning test; TMT = Trail making test; word = word reading; color = color naming; color-word = color-word interference; LPS = Leistungspruefsystem; BTA = brief test of attention; RWT = Regensburger Wortfluessigkeitstest; BDI: Beck’s depression index; PR = percentile rank. n.a.: ANOVA/Kruskal-Wallis test non-significant (*p* > 0.05).

Test	One-way ANOVA/Kruskal–Wallis test		p-value of post-hoc tests		
	F-value/H-value	p-value	ET1 vs. HC	ET2 vs. HC	ET1 vs. ET2
**MOCA [score]**	F_2,35_ = 2.106	0.137	n.a		
**MMSE [score]**	F_2,36_ = 1.299	0.285	n.a		
**CVLT [score]**					
**Immediate recall**	H(2) = 6.564	*0.038*	0.080	0.130	1.000
**Short delay recall**	H(2) = 5.919	0.052	n.a		
**Long delay recall**	H(2) = 5.743	0.057	n.a		
**Cued recall**	H(2) = 6.312	*0.043*	*0.045*	0.415	1.000
**Recognition**	H(2) = 2.747	0.253	n.a		
**TMT [PR]**					
**Part A**	H(2) = 8.929	*0.012*	0.137	*0.016*	0.655
**Part B**	H(2) = 8.637	*0.013*	*0.048*	*0.044*	1.000
**Stroop [sec]**					
**Word**	F_2,39_ = 6.709	*0.003*	*0.031*	*0.006*	0.807
**Color**	F_2,39_ = 4.127	*0.024*	0.127	*0.038*	1.000
**Color-word**	F_2,39_ = 4.137	*0.023*	0.101	*0.045*	1.000
**LPS [score]**					
**Subtest 7**	F_2,37_ = 1.346	0.273	n.a		
**Subtest 9**	F_2,37_ = 3.008	0.061	n.a		
**Block design [T-value]**	F_2,36_ = 3.657	*0.036*	0.059	0.202	1.000
**Complex Figure Test [score]**					
**Copy**	H(2) = 2.155	0.340	n.a		
**Delayed**	H(2) = 2.140	0.343	n.a		
**Digit span [PR]**					
**Forward**	H(2) = 8.696	*0.013*	0.177	*0.015*	0.62
**Backward**	H(2) = 9.805	*0.007*	*0.032*	*0.024*	1.000
**BTA [T-value]**	H(2) = 3.628	0.163	n.a		
**RWT [PR]**					
**Letter-cued**	H(2) = 4.313	0.116	n.a		
**Switching: letter-cued**	H(2) = 4.306	0.116	n.a		
**Category-cued**	H(2) = 13.927	< *0.001*	*0.022*	*0.002*	0.655
**Switching: category- cued**	H(2) = 12.036	*0.002*	*0.014*	*0.01*	1.000
**BDI [score]**	F_2,39_ = 6.614	*0.003*	*0.006*	1.000	*0.025*

### ETRP

**Fig. 2:**
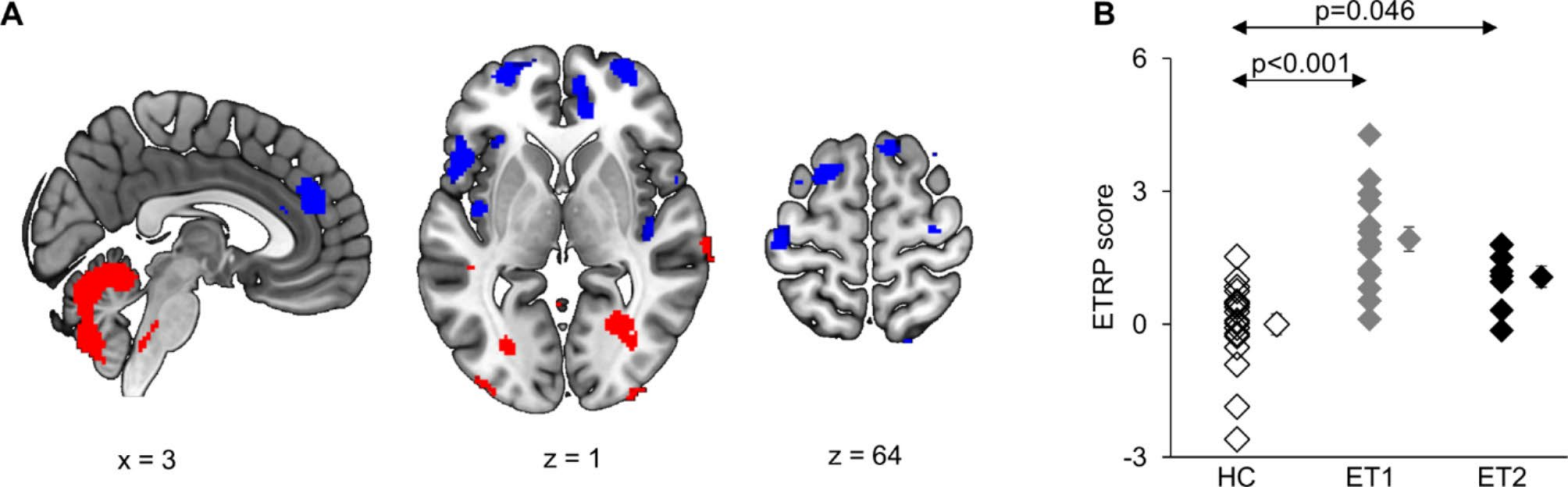
**(A)** Essential tremor related metabolic spatial covariance pattern (ETRP) identified in FDG-PET scans. Regions of relative metabolic increases are displayed in red, relative metabolic decreases in blue. Only regions surviving bootstrapping (1000 iterations) at a CI of 95% are displayed. Blobs are superimposed on a standard T1 template, coordinates are displayed in MNI standard space. **(B)** ETRP subject scores were significantly increased compared with HC in the derivation cohort (ET1) and an independent validation cohort (ET2). Error bars present the SEM. ET: essential tremor; HC: healthy controls; MNI: Montreal Neurological Institute; FDG-PET:^18^F-fluorodeoxyglucose positron emission tomography.

SSM/PCA revealed a stable spatial covariance pattern, i.e. ETRP. The first 9 PCs accounted for 52.2% of data variance. The linear combination of components 1, 3, 8 and 9 provided the best differentiation between ET patients and HC subjects, accounting for 26.8% of the variance (component 1: 14.3%, component 3: 5.8%, component 8: 3.6%, component 9: 3.1%). The ETRP was characterized by relatively increased metabolic activity in the cerebellum, brain stem, and temporo-occipital areas accompanied by relative hypometabolism mainly in fronto-temporal and parietal cortices, including the primary motor cortex (Fig. 2A).

ETRP expression differed significantly across groups (F(2,39) = 15.950, *p* < 0.001). Post-hoc tests showed higher scores in ET1 (derivation cohort; *p* < 0.001) and ET2 (validation cohort; *p* = 0.046) groups compared with HC. There was a trend towards lower scores in ET2 compared with ET1 subjects (*p* = 0.163) (Fig. 2B).

### Correlation of ETRP expression with tremor severity and cognition

**Fig. 3:**
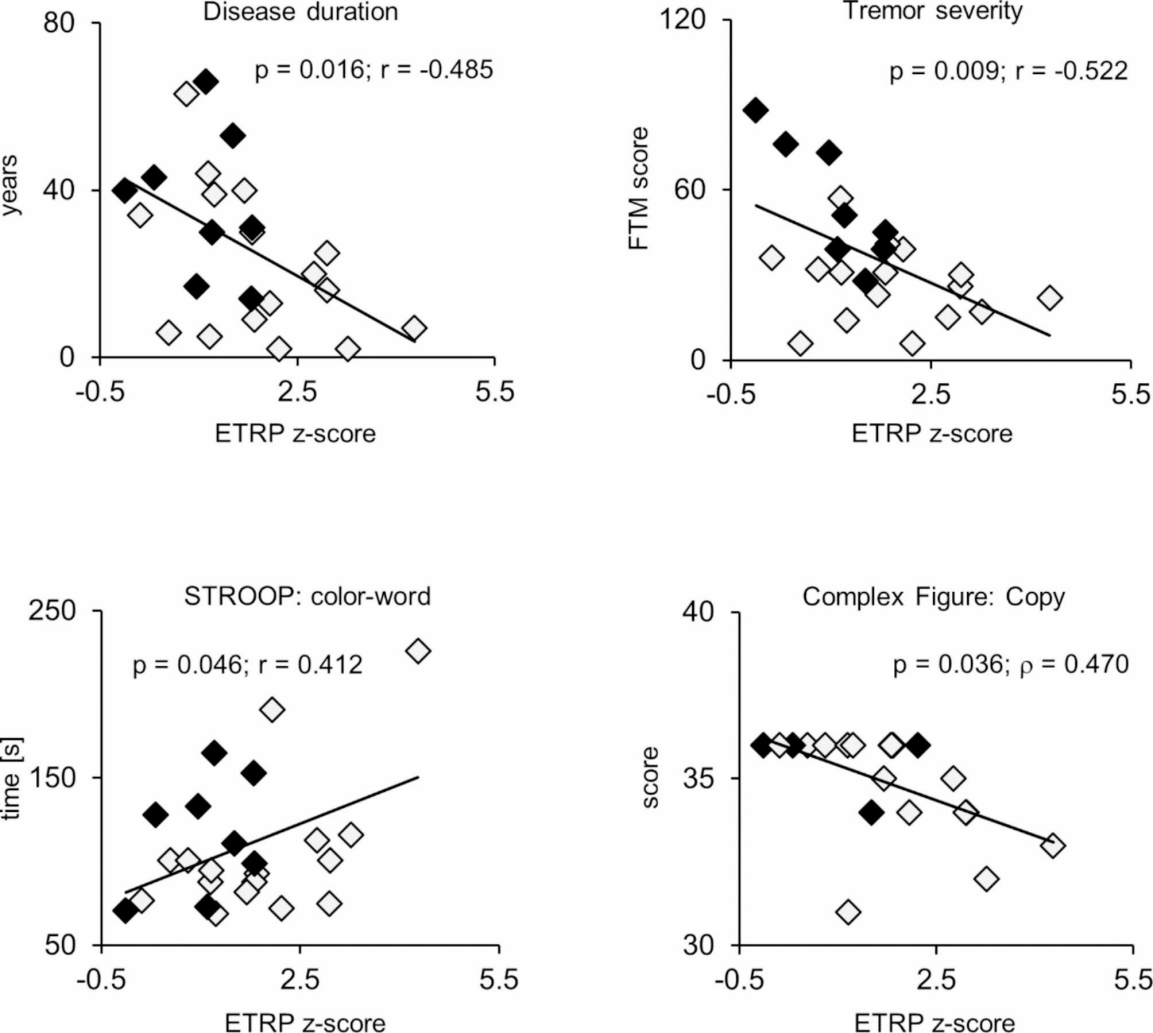
Correlations of disease duration, tremor severity, and cognitive performance with ETRP expression. ET1 subjects are displayed as gray diamonds, ET2 subjects are shown in black. ETRP: Essential tremor related metabolic spatial covariance pattern; FTM: Fahn-Tolosa-Marin scale, STROOP: Stroop test (color-word interference task); Complex figure: Complex figure test (copy task).

We found inverse correlations of ETRP expression with FTM scores (*r* = -0.522, *p* = 0.009) and disease duration (*r* = -0.485, *p* = 0.016) (Fig. 3). In contrast, higher network scores were associated with poorer cognitive performance as demonstrated by significant correlations of ETRP expression with the STROOP color-word (*r* = 0.412, *p* = 0.046) and complex figure tests (*r* = -0.541, *p* = 0.014) (Fig. 3). Because only the ET1 cohort was used for pattern derivation, we conducted separate analyses for the ET1 and ET2 cohorts, respectively. The correlation of FTM with ETRP expression remained significant in ET2 subjects (*r* = -0.867, *p* = 0.005). The correlation of ETRP expression with STROOP and complex figure test scores remained significant in the ET1 group (STROOP word: *r* = 0.535, *p* = 0.033; color: *r* = 0.535, *p* = 0.033; color-word: *r* = 0.546, *p* = 0.029; complex figure test: *r* = -0.617, *p* = 0.01). Additional correlations in the latter group were found between ETRP and CVLT scores (1. recall, ρ = -0.583, *p* = 0.023).

### Effect of DBS

DBS markedly improved tremor in ET2 subjects (FTM OFF 54.9 ± 21.4 vs. ON 22.8 ± 19.8, *p* < 0.001). There were few effects on cognitive performance. Active stimulation worsened MMSE (OFF 28.20 ± 1.10 vs. ON 27.20 ± 1.64, *p* = 0.034) and RWT (letter-cued task) performance (OFF 44.13 ± 26.86 vs. ON 32.00 ± 29.16, *p* = 0.021). In contrast, better performance with the DBS turned ON was achieved on the cued-recall task of the CVLT (OFF 4.25 ± 3.85 vs. ON 5.63 ± 3.20, *p* = 0.042). There was no effect of DBS on the remaining cognitive tests (*p* > 0.1). DBS did not alter ETRP expression at the group level (Z-Score OFF 1.07 ± 0.69 vs. ON 1.28 ± 0.60, *p* = 0.231). However, inspection of individual values showed an increase of ETRP expression in all but one subject in the ON state (supplementary Fig. 1). Given the weak modulatory effect on network expression, we performed additional SPM based voxel-wise post-hoc analyses of regional metabolism in regions of interest (ROI) encompassing the major ETRP nodes. We used the WFU Pickatlas^32,33^ to delineate the ROI. The following regions were selected: cerebellum and brainstem encompassing the “red nodes”, and bilateral superior frontal gyrus, bilateral insular cortex, bilateral anterior cingulate cortex, and bilateral precentral gyrus which encompassed the “blue” network nodes (supplementary Fig. 2). The voxel threshold was set at *p* < 0.001 (FWE corrected at *p* < 0.05, cluster extent threshold: 20 voxels). We did not observe DBS induced changes for neither contrast (OFF > ON and OFF < ON, respectively) in none of the ROI.

### Relationship of ETRP with PDRP

Computation of PDRP expression did not reveal any difference among ET1, ET2, and HC groups (F(2,39) = 0.393, *p* = 0.678). However, the correlation of ETRP with PDRP expression was significant (*r* = 0.687, *p* < 0.001). Furthermore, there was a very weak, albeit significant correlation of PDRP and ETRP voxel-weights (r^2^ = 0.027, *p* < 0.001).

## Discussion

For the first time, we have identified a metabolic spatial covariance pattern characterizing ET. The ETRP was comprised of brain regions such as the cerebellum, brain stem, and motor cortices that commonly have been associated with tremor genesis. The ETRP also included brain regions not involved in postulated concepts of tremor propagation, but which have been associated with cognitive dysfunction in ET. Along these lines, our data confirm previous reports of cognitive impairment in ET. ETRP expression exhibited negative correlations with disease duration and tremor severity, suggesting a primarily compensatory role of the network that fails in advanced disease stages. The inverse correlation of network expression with cognitive function, i.e. higher network expression in more severely impaired subjects, indicates that the network’s role differs for cognitive and motor domains. While DBS markedly improved tremor, we observed only marginal and inconsistent effects on cognitive performance. There was no ETRP modulation at the group level, albeit ETRP expression increased in seven out of 8 subjects in the ON condition, supporting the hypothesis of a potential compensatory role of this network.

The widely accepted concept of tremor genesis postulates that the cerebellum is a key driver of aberrant oscillatory activity in a tremor network comprising the cerebellum, brainstem, thalamus und (pre)motor cortices^4^. Along these lines, numerous functional MRI studies have reported aberrant cerebellar-thalamico-cortical connectivity in the resting state in ET^11^. A consistent finding was increased cerebello-thalamico connectivity that was positively correlated with clinical tremor severity^34,35^. In contrast, intracerebellar connectivity and cerebello-(motor)cortical projections seem to be reduced in ET and show negative correlations with tremor severity^36,37^, suggesting that the cerebellum predominantly facilitates tremor through thalamico-cortical projections^38^. It remains unclear how these complex changes of cerebellar connectivity translate into corresponding changes of brain perfusion and metabolism. Whereas early studies reported increased cerebral blood flow in the cerebellum^39^, these findings have not been confirmed by more recently published work^40^. Few studies have used FDG-PET to quantitatively explore ET related brain metabolism^11^. Surprisingly, one study reported reduced cerebellar metabolism in ET patients compared to HC. However, the authors used a liberal threshold without correcting for multiple comparisons in their voxelwise analysis^41^. Other studies did not reveal changes of cerebellar metabolism, but inconsistent decreases of wide spread cortical regions, and increased activity in the thalamus in one study^42,43^. The most prominent hypermetabolic node was situated in the cerebellum in our study, supporting a pivotal role of the cerebellum in ET pathology. The obvious explanation for increased cerebellar activity would be an aberrant over-activation driving tremor genesis. However, the inverse correlation with tremor severity and disease duration with network expression implies that cerebellar hypermetabolism may be a temporary rather than consistent phenomenon that primarily serves a compensatory role that fails over time. Along these lines, there is evidence for structural remodeling of the cerebellum in early onset ET suggesting compensatory processes^44,45^. The role of relatively increased temporo-occipital metabolism is unclear. Verger and colleagues reported increased activity in a similar region in in a cohort of ET patients that decreased in patients with a good treatment response to therapeutic thalamic gamma knife surgery^46^. Lastly, we found reduced metabolism in the primary motor cortex which may reflect reduced cerebello-cortical input^34^.

ET subjects performed worse compared to HC in various neuropsychological tests most of which relate to executive function, working memory, and verbal fluency. These findings are in line with previous reports of deficits in attention and executive domains^7,47,48^. The pathophysiological underpinnings of cognitive decline in ET remain elusive. It has been hypothesized that alterations of cerebello-cortical connectivity may facilitate executive dysfunction in ET^9^. The prefrontal cortex, and dorsolateral prefrontal cortex in particular, have consistently been shown to exhibit aberrant connectivity with various cortical regions, including parietal, cingulate, and insular cortices in ET^49^. It is unclear, however, if frontal lobe dysfunction is an independent contributor to cognitive decline in ET or rather a consequence of altered cerebellar input^50^. That said, findings from structural imaging studies showing volume loss in wide-spread areas including the insular/parahippocampal cortices, cingulate cortex, caudate nucleus, and prefrontal cortex make it seem unlikely that cerebellar dysfunction is the sole basis of cognitive impairment^10,51^. These patterns of altered connectivity and atrophy in frontotemporal and cingulate cortices show considerable overlap with the “blue” ETRP nodes. In contrast to correlations of network expression with tremor severity, we found higher network scores in cognitively impaired individuals compared to those with intact cognition. This implies that the ETRP likely occupies different roles in motor and cognitive deficits, respectively. Whereas increased ETRP activity was pronounced in subjects with only mild tremor and appeared to break down in later disease stages, there was a linear increase of network activity with declining cognitive performance with no evidence of a ceiling effect. Of note, there was an only marginal correlation of FTM scores and measures of cognitive performance, indicating that the neuronal correlates of tremor genesis and cognitive dysfunction are not identical. In line with our finding, the emergence of cognitive impairment has been found to be related to older age at onset, but seems to be largely independent of tremor severity^50^.

It has repeatedly been suggested that PD and ET may share some pathophysiological properties^52^. For example, some authors have reported slightly reduced striatal dopaminergic integrity^53^, Lewy bodies have been found in the brains of ET individuals^54^, and subjects with long lasting ET are at increased risk to develop PD^52^. That said, other studies have reported controversial results^55^, and there is no proven pathophysiological link between ET and PD. While an abnormal brain network specifically associated with PD tremor (PDTP) has been identified previously^56^, this network was derived from a very small cohort of only 9 patients and has not yet been reproduced in independent cohorts. Furthermore, while PDTP expression correlates with clinical measures of tremor severity, it is not associated with other PD motor symptoms such as rigidity and akinesia^56^. In contrast, the PDRP is an well-established, SSM based metabolic network that has proven itself to reliably reflect PD phenotype and pathophysiology^12^, which is why we chose this network for comparative analyses with the ETRP.

Prospectively computed PDRP scores in ET individuals in our study did not differ from HC. However, there was a positive correlation of PDRP and ETRP expression. That said, there was an only marginal overlap of PDRP and ETRP voxel weights. In fact, less than 3% of the variance of the PDRP voxel weights was accounted for by corresponding values of the ETRP. This is well below r-values observed in previous studies applying similar correlation routines to compare the topographical overlap of patterns regarded as distinct from the PDRP, such as networks specific to multiple system atrophy or normal movement^57,58^. Furthermore, our algorithm did not include procedures to correct for autocorrelation effects, the latter of which may lead to false significant p-values in spite of very small r-values^57^. Thus, it appears unlikely that PD and ET share substantial overlap in terms of their metabolic topography. As longitudinal data are lacking, it remains unclear if the significant relationship of network scores may hint towards an increased risk to subsequently develop PD as suggested by epidemiological studies in ET^52^.

Previous studies in PD patients have shown significant effects of DBS on PDRP expression that were accompanied by concurrent improvements of motor function in the ON compared with OFF state^59,60^. We expected similar effects of DBS on ETRP expression, particularly given the marked improvement of tremor in the stimulation ON condition. However, there were no significant stimulation induced changes of ETRP expression at the group level. That said, an inspection of individual data revealed that all but one ET2 subjects showed higher ETRP expression in the ON compared with OFF condition. This would strengthen the hypothesis of a primarily compensatory role of the ETRP that is enhanced by DBS. It is conceivable that the small number of subjects in the ET2 cohort was too small to discern effects on ETRP modulation at the group level. Along these lines, additional ROI based analyses did not reveal DBS induced changes at the regional level either. Alternatively, DBS related improvement of tremor may have been mediated by regional effects outside the ETRP. A recently published functional MRI suggested that DBS has a modulatory effect on cerebello-thalamico-cortical circuitry^61^. Specifically, the authors reported increased resting sate activity in the premotor cortex in the ON vs. OFF condition^61^. However, the same group did not observe significant effects of DBS of the caudal zona inserta on resting state brain connectivity in another study including 13 ET patients compared with matched controls, despite marked improvement of tremor scores in the ON state. The authors argued that modulation of cerebello-thalamic-cortical may be task dependent^62^ To our knowledge, only two studies have explored DBS effects on brain metabolism using FDG-PET^63,64^. Both studies reported increased thalamic activity in the ON vs. OFF condition, one study found additionally increased activity in the cerebellar dentate nuclei^64^. There are possible explanations for the divergent findings compared with of our study. Firstly, we utilized a multivariate analytical network approach compared to univariate voxel- or region of interest-based analyses in the previous studies, and the ROI included in our secondary post-hoc analyses differed from those investigated in the previous studies. Secondly, we limited the stimulation OFF time to 12 h, whereas the stimulator was turned off 72 h in the other studies. Even though tremor in ET patients usually reemerges rather instantaneously after the stimulator is turned off, the 12 h interval may not have been sufficient to allow for the complete cessation of stimulation induced effects on brain metabolism.

We found small and inconsistent effects of DBS on cognitive performance. Bilateral DBS of the subthalamic nucleus can deteriorate cognitive function in PD^65^. In contrast, deleterious effects of DBS on cognition in ET individuals are rare^66^. Some studies even have suggested a slight improvement of cognitive performance following unilateral DBS^67^. However, most studies did not report significant effects of DBS on cognition in ET patients^68,69^. Alongside the divergent correlation of ETRP expression with ET motor and cognitive symptoms, the contrasting effects of DBS on motor and cognitive domains indicate distinct neuronal underpinnings of tremor and cognitive impairment in ET.

While the inverse correlation of network expression with disease duration and tremor severity my appear counter intuitive, the significant correlations with both motor and cognitive markers suggest an association of the ETRP with disease phenotype. Nevertheless, a correlation obviously does not imply causality, and decreasing ETRP expression over time clearly limits its utility as an biomarker. However, decreasing pattern expression of disease specific spatial covariance patterns is not unique to the ETRP. As an example, waning network activity related to REM sleep behavior disorder (RBD), a condition strongly predictive of the later development of PD, was found in RBD subjects approaching motor onset, whereas PDRP expression increased. This observation suggests that changes of network expression are not necessarily linear, and dynamic modelling of network architecture during the course of the disease may occur^70^. Unsurprisingly, DBS patients were more severely affected and had a longer disease duration compared with ET1 subjects. Thus, albeit not significantly, ETRP expression was lower in the latter cohort. However, the lower scores in ET2 individuals could also partially be caused by a derivation bias, as ET2 subjects were not part of pattern identification and no independent control cohort was available for z-scoring. That said, using ET1 subjects, OFF stimulation scans of ET2 subjects, and HC for pattern derivation resulted in a stable pattern with a very similar topography as the original ETRP (supplementary Fig. 3). Indeed, the ETRP nodes appeared to be more robust as indicated by the larger spatial extent at a 95% confidence interval. There was a strong correlation of voxel weights between the two networks, accounting for 69% of the respective variance (*r* = 0.833, *p* < 0.001). This observation indicates that the ETRP was not driven by mildly affected subjects and, furthermore, that the ETRP not solely reflects the clinical phenotype, but also disease traits not captured by clinical assessments. Finally, ET is a very heterogeneous disease in terms of clinical presentation, disease progression, and epidemiology. This translates into equivocal findings reported by functional, structural, and metabolic imaging studies^11^. Future studies in larger cohorts are warranted to confirm whether ET indeed can be characterized by a uniform metabolic topography.

Our study is not without limitations. The sample size was small. This was particularly true for ET2 subjects, which may have hampered the identification of DBS mediated effects on network expression and limited statistical power of correlational analyses. Thus, the results of our exploratory study have to be interpreted with caution. Moreover, the study was cross-sectional, and longitudinal data are needed to confirm the relationship of ETRP expression with clinical disease markers. Some ET subjects, particularly those with DBS, were severely affected and not able to complete all cognitive tasks which poses a potential bias to our analyses, and the magnitude of cognitive impairment was not homogeneous across subjects. Lastly, several patients were on ET specific medication. To our knowledge, there are no studies that have explored the effect of primidone or propranolol on brain metabolism, but propranolol has been shown to alter cerebral blood flow in a harmaline rat model of ET^71^. We are not aware of similar studies investigating primidone, but a previous MRI study at least did not suggest a significant impact of primidone on gamma-amino-butyric acid metabolism in ET^72^. Of note, medicated ET1 subjects did not differ from drug naive individuals in terms of ETRP expression (*p* = 0.806) or FTM scores (*p* = 0.888). Thus, even though we cannot exclude medication related effects, it is unlikely there was a major impact on brain metabolism.

In conclusion, we identified an ET specific metabolic spatial covariance network. The significant correlations with tremor severity, disease duration, and cognitive performance indicate that the network is related to ET pathophysiology, but do not imply causality. The distinct correlations of network scores with motor and cognitive symptoms suggest that the neural correlates underlying tremor and cognitive impairment are not interchangeable.

## Electronic supplementary material

Below is the link to the electronic supplementary material.

Supplementary Material 1

## Data Availability

Data used for the preparation of this article will be made available upon reasonable request to the corresponding author within the legal and ethical regulations as demanded by the local authorities.
