# Supplementary material for: Identification of a metabolic brain network characterizing essential tremor
Source: Sci Rep. 2025 Jan 16;15:2138. doi: 10.1038/s41598-024-82069-4 (PMC11739557; doi:10.1038/s41598-024-82069-4)
Supplement: Supplementary file 1 — Supplementary Material 1 [file 41598_2024_82069_MOESM1_ESM.pdf]

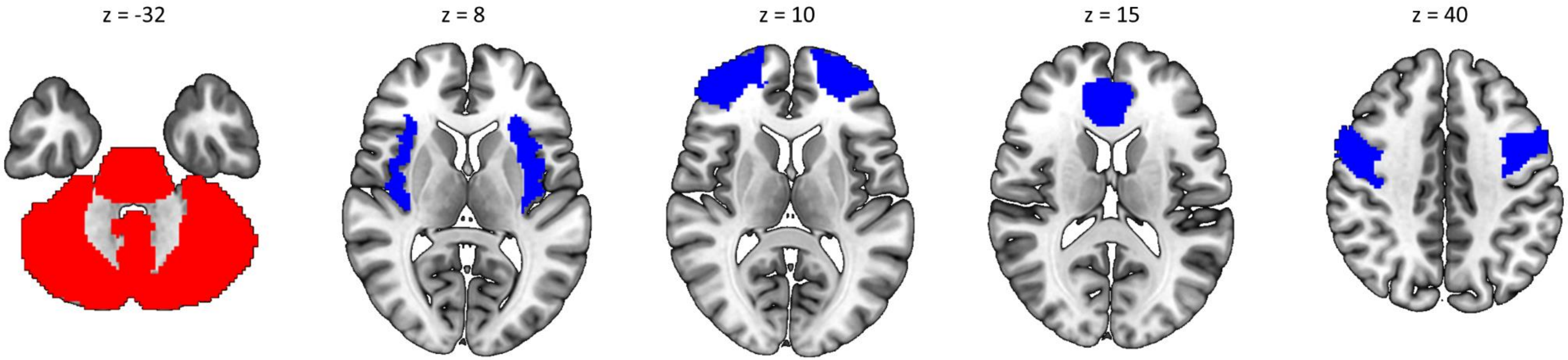

Sppl. Fig. 1: Display of regions of interest (ROI) used for univariate voxel-wise analyses of DBS effects. Red blobs encompass hypermetabolic ETRP nodes, blue blobs hypometabolic nodes. Coordinates are displayed in Montreal Neurological Institute (MNI) standard space. ROI were superimposed on a standard MRI T1 template. DBS: Deep brain stimulation, ETRP: Essential tremor related metabolic spatial covariance pattern.

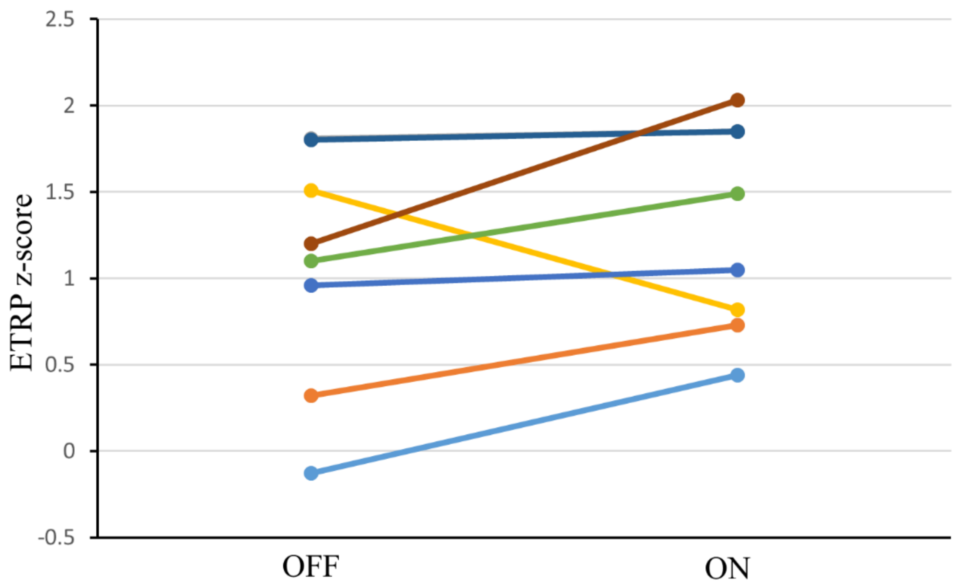

Sppl. Fig. 2: Individual changes of ETRP expression in 8 ET subjects with thalamic deep brain stimulation. Network expression increased in all but one subject when stimulation was turned on. ETRP: Essential tremor related metabolic spatial covariance pattern; OFF: Stimulation turned off; ON: Stimulation turned on; ET: Essential tremor.

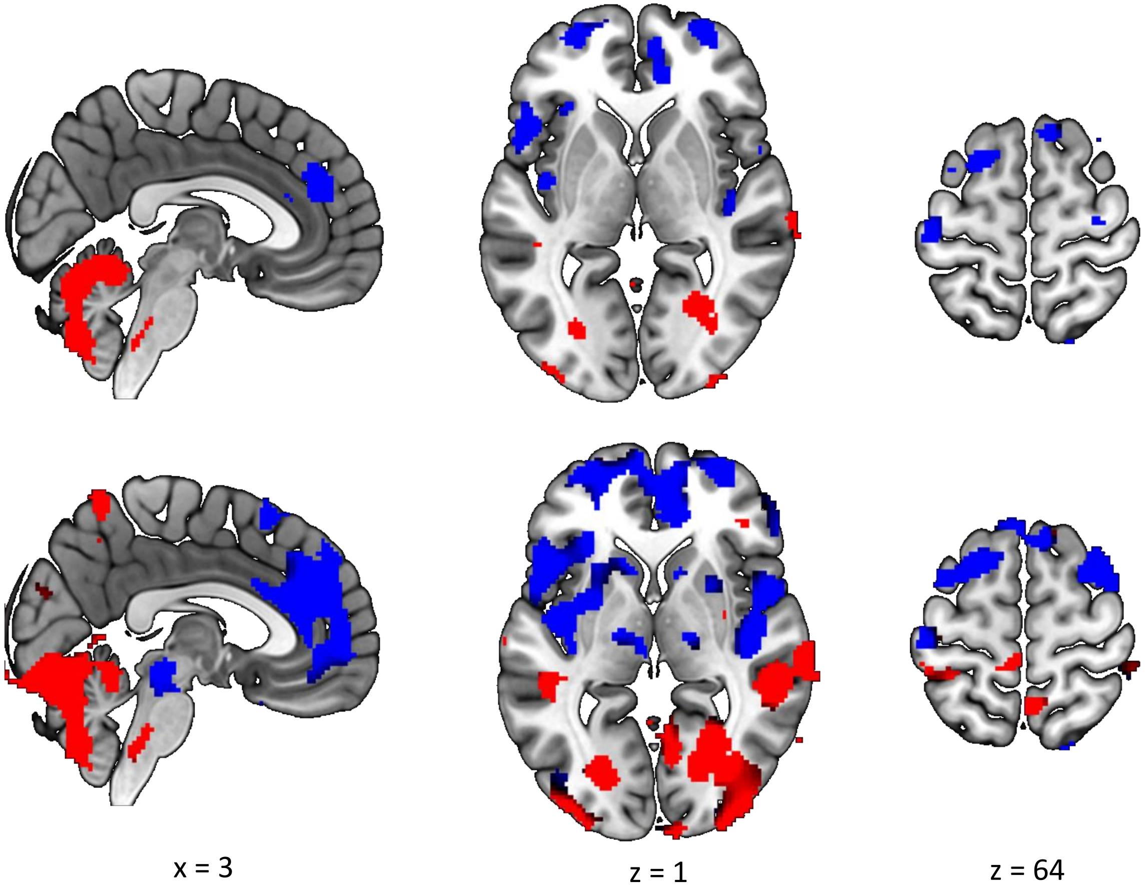

Sppl Fig. 3: Display of ETRP topography. *Top panels* ETRP derived from ET1 subjects and HC. *Bottom panels* ETRP derived from combined ET1/ET2 (OFF condition) subjects and HC. Red blobs represent regions of relative metabolic increase, blue blobs regions of relative metabolic decrease. Coordinates are displayed in Montreal Neurological Institute (MNI) standard space. Blobs were superimposed on a T1 MRI standard template. ETRP: Essential tremor related metabolic spatial covariance pattern; ET1: Essential tremor cohort 1; ET2: Essential tremor cohort 2; HC: Healthy controls.
